# Impacts of Co-Existing Chronic Rhinosinusitis on Disease Severity and Risks of Exacerbations in Chinese Adults with Bronchiectasis

**DOI:** 10.1371/journal.pone.0137348

**Published:** 2015-09-04

**Authors:** Wei-jie Guan, Yong-hua Gao, Hui-min Li, Jing-jing Yuan, Rong-chang Chen, Nan-shan Zhong

**Affiliations:** 1 State Key Laboratory of Respiratory Disease, National Clinical Research center for Respiratory Disease, Guangzhou Institute of Respiratory Disease, First Affiliated Hospital of Guangzhou Medical University, Guangzhou Medical University, Guangzhou, Guangdong, China; 2 Department of Respiratory and Critical Care Medicine, First Affiliated Hospital of Zhengzhou University, Zhengzhou, Henan, China; University of Dundee, UNITED KINGDOM

## Abstract

**Background:**

Mounting evidence supports the notion of “one airway, one disease.”

**Objective:**

To determine whether chronic rhinosinusitis (CRS) poses adverse impacts on Chinese adults with bronchiectasis.

**Methods:**

We enrolled 148 consecutive adults with clinically stable bronchiectasis. CRS diagnosed based on the 2012 EP^3^OS criteria. We systematically evaluated the bronchiectasis etiology, radiology, lung function, sputum bacteriology, airway inflammatory biomarkers, *Bronchiectasis Severity Index*, cough sensitivity and healthcare resource utilization. All patients were prospectively followed-up for 1 year to examine the frequency of bronchiectasis exacerbations (BEs).

**Results:**

Forty-seven patients (31.8%) were diagnosed as having CRS. Bronchiectasis etiologies did not vary statistically between CRS and no-CRS group. There was a trend towards non-statistically higher *Bronchiectasis Severity Index* [6.4±3.4 vs. 5.0(6.0), P = 0.19], a higher proportion of patients with BEs needing hospitalization before enrollment (48.9% vs. 29.7%, P = 0.13), poorer FVC [78.2±19.8% vs. 82.2(16.8)%, P = 0.54] and FEV_1_ [68.2±24.8% vs. 74.8(21.2)%, P = 0.29], a higher prevalence of *Pseudomonas aeruginosa* isolated (36.2% vs. 26.7%, P = 0.27) or colonized in sputum (36.2% vs. 21.8%, P = 0.12) and greater capsaicin cough sensitivity [C2: 3.9(123.0) μmol/L vs. 11.7(123.0) μmol/L, P = 0.81; C5: 62.5(996.0) μmol/L vs. 250.0(973.0) μmol/L, P = 0.32]. Patients with CRS had significantly greater risks of experiencing BEs during follow-up (P = 0.02 for negative binominal regression test).

**Conclusion:**

Chinese adults with bronchiectasis appear to have a lower prevalence of CRS than that in western countries. There was a trend towards greater adverse impacts on bronchiectasis in patients with CRS. Studies with greater sample sizes might help to resolve this issue. In future clinical practice, physicians should be vigilant to the screening of concomitant CRS in bronchiectasis so as to better improve patient’s healthcare. Our findings may be of clinical significance in that proper treatment of upper airway symptoms due to CRS will be the prevention of infection or re-infection of the tracheobronchial tree, which should be addressed for the future management of bronchiectasis.

## Introduction

It has become increasingly recognized that the upper and lower airways are indeed united [1]. Some respiratory diseases may co-exist with upper airway disorders which mutually interact, leading to greater illnesses. For instance, apart from a higher prevalence of allergic rhinitis in patients with asthma [1], recent studies have further shown that chronic rhinosinusitis (CRS) was fairly common in patients with bronchiectasis [2] and posed adverse impacts on lung function [2], quality of life [3,4] and the lost sense of smell [4,5]. The mechanisms responsible for these adverse impacts of CRS on bronchiectasis remain unclear, but might be associated with impaired ciliary ultrastructures and ciliary dysfunction [6,7].

Bronchiectasis is a chronic airway disease with permanently dilated small- and medium-sized airways which are associated with the vicious cycle of airway infection, inflammation and destruction [8–11]. These events lead to impaired lung function [12,13] and quality of life [14] and recurrent infective exacerbations [15]. Concurrent management approaches mainly comprise the management of infective exacerbations [15,16], and the administration of antibiotics [17,18], anti-inflammatory therapy [19,20], mucolytics [21,22], chest physiotherapy and postural drainage [23]. However, none of these approaches addressed sufficiently the management of upper airway inflammatory diseases such as CRS, a common comorbid disease, in patients with bronchiectasis. It is unclear whether the management of CRS would be needed for the treatment of bronchiectasis in the oriental Asian population.

The Guangzhou Bronchiectasis Study, starting from September 2012, has offered an opportunity to examine the association of CRS and bronchiectasis in Chinese adults. Since CRS in bronchiectasis has been characterized [2–5], this study focused on the effects of CRS on the lower airways symptoms, chest imaging abnormality, lung function and future risks of exacerbations.

## Methods

### Subjects

Adults with bronchiectasis were consecutively recruited from our out-patient respiratory clinics. Bronchiectasis was diagnosed according to chest high-resolution computed tomography (HRCT) which was performed within 12 months. Eligibility criterion was clinical stability, which was defined as free of bronchiectasis exacerbations (BEs) for at least 4 weeks. BEs denoted 3 or more items lasting for 24 hours or longer: increased cough frequency; increased sputum volume or purulence; dyspnea; exercise intolerance; hemoptysis; T>37.5°C; significantly increased crackles on auscultation; increased pulmonary infiltration (chest X-ray) [10,13]. Patients with malignancy, upper respiratory tract infection or antibiotic use in the previous 4 weeks were excluded. None of the patients had ever used inhaled antibiotics in this study.

The Ethics Committee of First Affiliated Hospital of Guangzhou Medical University gave ethics approval [approval No.: Medical Ethics Year 2012 (The 33^rd^)]. Prior to enrollment, all patients gave written informed consent which has been approved by the Ethics Committee. All informed consent forms were kept by the investigators, in separate patient document files.

### Diagnosis of CRS and bronchiectasis

CRS was diagnosed based on the EP^3^OS criteria [24], which included:

1. Two or more symptoms reflecting nasal and paranasal sinuses inflammation, lasting for at least 12 weeks, without complete resolution: nasal blockage/obstruction/congestion or nasal discharge (anterior/posterior nasal drip), plus facial pain/pressure, or plus reduction/loss of smell.
2. CT changes (mucosal changes of ostiomeatal complex and/or sinuses).

The presence/absence of nasal polyps could not be ascertained because nasal endoscopy or tissue biopsy was not performed.

Because only a minority of patients had received regular treatment of CRS, we were unable to compare the pre- and post-treatment differences in various clinical variables.

Chest HRCT at collimation of 2mm or less, within 12 months, was assessed. Bronchiectasis was diagnosed if any of the following criterion was met: 1) the internal diameter of bronchi was greater than accompanying pulmonary artery; 2) normal bronchial tapering along travel on sequential slices is lacking; 3) presence of visible bronchi within 10 mm to the pleura [10,13].

### Clinical assessments

Details regarding on the methods for determining the etiology [25], airway and systemic inflammation [10], sputum bacteriology [10], BEs [13], lung function [13,25] and capsaicin cough sensitivity [26] have been published previously.

Briefly, patients completed the questionnaires which contained the information regarding the demographics, symptoms, medical and smoking history, and the use of healthcare resources. Underlying causes of bronchiectasis were systematically evaluated according to our proposed flowchart [25]. Chest HRCT score (modified Reiff score) was determined by one experienced radiologist (> 10 years of experience) who was masked to patient’s grouping. Spirometry and diffusing capacity were measured or performed following medication withdrawal [10]. Within 2 hours of sampling during the baseline hospital visit, fresh sputum was immediately sent for microbiology assessment [10]. Cough sensitivity were examined by using capsaicin cough challenge test [26]. The severity of bronchiectasis was determined by using the *Bronchiectasis Severity Index* (BSI), which is an accepted composite score that takes into account the age, body-mass index, the number of bronchiectatic lobes, *Pseudomonas aeruginosa* infection, colonization of other pathogenic microorganisms, FEV_1_% predicted, dyspnea, and the number of hospitalization and exacerbations in the previous year [27].

Patients were prospectively followed-up for 1-year, at 3-month hospital visits. We meticulously recorded the BEs. Discrepancies of decisions, if any, were adjudicated following discussion (W.J.G. and Y.H.G.).

### Statistical analysis

Data on the prevalence of CRS in bronchiectasis have been extremely scarce. To our knowledge, apart from the serial studies by Guilemany et al [2–5], there has been no study that mentioned the prevalence of CRS in patients with bronchiectasis, particularly in the Chinese cohort. There is no study that has investigated the annual incidence of bronchiectasis exacerbations in patients with CRS. Furthermore, the prevalence of CRS among bronchiectasis patients in our cohort (see RESULTS section) differed considerably from that in Guilemany et al’s study [2]. Therefore, we could not calculate the sample size for this study. However, according to our previous experience and the studies by Guilemany et al [2–5], the sample size of 148 patients might have been suitable for the single center study.

Data were analyzed by using Graphpad Prism 5.0 (Graphpad Inc., USA) and SPSS 16.0 (SPSS Inc., Ill, USA). Numerical data were presented as mean ± standard deviation, or median (interquartile range) as appropriate. Two-group comparisons were made by using independent t-test or Mann-Whitney test. The rates between two groups were compared by using chi-square test. The distribution of BEs within 2 years prior to recruitment, and during the 1-year follow-up, was plotted in bar charts. The association between CRS and the BEs before recruitment were analyzed initially by using univariable Logistic regression model, followed by step-wise adjustment with the potential confounders (sex, smoking status, the diagnosis of asthma or COPD, and the BSI) based on multivariable Logistic regression model. The odds ratio (OR) and 95% confidence interval were reported for all risk estimates. The BEs during 1-year follow-up were analyzed with Cox regression model, with sex, smoking status, the diagnosis of asthma or COPD, and the BSI as covariates. We considered *P*<0.05 to be statistically significant for all comparisons.

## Results

### Subject recruitment

Of 160 patients, 12 (7.5%) withdrew consent during screening. Of the remaining 148 patients who were further screened, 47 (31.8%) were diagnosed as having CRS. 24 patients (22.2%) were still included in survival analyses despite lost to follow-up at month 6 or thereafter following recruitment.

### Baseline clinical characteristics

Baseline characteristics are displayed in Table 1. Our findings were less likely to suffer from selection bias, since the socioeconomic status (whole family’s income within the previous 1 year) was overall normally distributed [25].

**Table 1:** Clinical characteristics of patients with bronchiectasis.

Parameters	CRS	No CRS	P value
**No. of patients**	47	101	-
**Age (years)**	43.6±14.8	45.0±13.4	0.58
**Height (cm)**	160.1±8.0	161.6±7.6	0.27
**Weight (kg)**	50.0 (11.5)	52.0 (10.8)	0.40
**Females (No., %)**	33 (70.2%)	59 (58.4%)	0.17
**Body mass index (kg/m** ^**2**^ **)**	20.4±2.7	20.0 (4.3)	0.80
**Body mass index <18.5 (No., %)**	11 (23.4%)	33 (32.7%)	0.25
**Age of bronchiectasis symptom onset (years)**	30.3±16.1	30.7±17.1	0.91
**Symptom onset for >10 years (No., %)**	19 (40.4%)	40 (39.6%)	0.92
**Duration of symptom onset (years)**	13.3±9.7	14.3±14.2	0.65
**24-hour sputum volume (ml)**	20.0 (35.0)	20.0 (25.0)	0.44
***Bronchiectasis Severity Index***	6.4±3.4	5.0 (6.0)	0.19
**Never-smokers (No., %)**	39 (83.0%)	92 (91.1%)	0.15
**Underlying causes**			
**Post-infectious (No., %)**	10 (21.3%)	30 (29.7%)	0.28
**Other known causes (No., %)** *	**18 (38.3%)**	**22 (21.8%)**	**0.04**
**Idiopathic (No., %)**	19 (40.4%)	49 (48.5%)	0.36

Numerical data were presented as mean ± standard deviation for normal distribution or otherwise median (interquartile range). Categorical data were expressed as number (percentage).

Data in bold indicated the comparisons with statistical significance.

* Of all bronchiectasis patients, other known causes consisted of immunodeficiency (n = 13, 8.8%), asthma (n = 8, 5.4%), gastroesophageal reflux (n = 6, 4.1%), aspergillosis (n = 2, 1.4%), rheumatoid arthritis (n = 2, 1.4%), Kartagener syndrome (n = 2, 1.4%), lung maldevelopment (n = 2, 1.4%), Young’s syndrome (n = 1, 0.7%), COPD (n = 1, 0.7%), lung sequestration (n = 1, 0.7%), yellow nail syndrome (n = 1, 0.7%) and diffuse panbronchiolitis (n = 1, 0.7%).

None of the patients had ever used inhaled antibiotics in this study.

The mean age was 43.6 years and 45.0 years in CRS and no-CRS group (P = 0.58), respectively. Females accounted for a non-significantly greater proportion in the CRS group (70.2% vs. 58.4%, P = 0.17). There were no statistically significant differences in the body-mass index (P = 0.80), the age of symptom onset (P = 0.91), the duration of symptom onset (P = 0.65), 24-hour sputum volume (P = 0.44), the *BSI* (P = 0.19) and the proportion of never-smokers (P = 0.15). The most common underlying cause was idiopathic (40.4% in CRS group vs. 48.5% in no-CRS group, P = 0.36), followed by other known causes (38.3% in CRS group vs. 21.8% in no-CRS group, P = 0.04) and post-infectious bronchiectasis (21.3% in CRS group vs. 29.7% in no-CRS group, P = 0.28).

### Medication use, healthcare utilization and comorbidities

The medications ever used within 6 months prior to enrollment, which primarily consisted of any antibiotics (P = 0.13), macrolides (P = 0.99), inhaled corticosteroids (P = 0.28) and mucolytics (P = 0.87), were similar between the two groups. There was a trend towards more frequent use of oral antibiotics in CRS group despite the non-statistically significant differences (P = 0.17).

The heathcare resource utilization was also comparable between the two groups, despite a non-significantly higher proportion of patients with BEs needing hospitalization [n = 23 (48.9%) vs. n = 30 (29.7%), P = 0.13] and a lower proportion of patients with BEs needing oral antibiotics only, without hospitalization, within the previous 2 years [n = 17 (36.2%) vs. n = 51 (50.5%), P = 0.13] in the CRS group. This could be partially reflected by the comparable frequency of BEs within the previous 2 years (median: 3.0 vs. 3.0, P = 0.46).

Apart from anxiety (34.0% vs. 38.6%, P = 0.59) and depression (31.9% vs. 26.7%, P = 0.52), other systemic diseases (i.e. hypertension, rheumatoid arthritis) were relatively infrequent in both groups (8.5% vs. 10.0%, P = 0.97). (Table 2)

**Table 2:** Comparisons of medications used within 6 months, healthcare utilization and comorbidities between the two group.

Parameters	CRS	No CRS	P value
**No. of patients**	47	101	-
**Antibiotics use (No., %)**	-	-	0.13
** None**	13 (27.7%)	44 (43.6%)	-
** Oral only**	27 (57.4%)	41 (40.6%)	-
** Systemic**	7 (14.9%)	16 (15.8%)	-
**Inhaled corticosteroid use (No., %)**	12 (25.5%)	18 (17.8%)	0.28
**Macrolides use (No., %)**	20 (42.6%)	43 (42.6%)	0.99
**Mucolytics use (No., %)**	35 (74.5%)	74 (73.3%)	0.87
**No. of BEs within 2 years**	3.0 (3.0)	3.0 (4.0)	0.46
**Healthcare utilization within 2 years (No., %)**	-	-	0.17
**No treatment of BEs** *	2 (4.3%)	7 (6.9%)*	-
** BEs needing oral antibiotics only without hospitalization**	17 (36.2%)	51 (50.5%)	-
** BEs needing systemic antibiotics without hospitalization**	5 (10.6%)	11 (10.9%)	-
** BEs needing hospitalization**	23 (48.9%)	30 (29.7%)	-
**Comobidities (No., %)**	-	-	-
** Anxiety** *	16 (34.0%)	39 (38.6%)	0.59
** Depression** *	15 (31.9%)	27 (26.7%)	0.52
** Other systemic diseases**	4 (8.5%)	10 (10.0%)	0.97

BEs: bronchiectasis exacerbations

* 2 patients without CRS had missing data.

Numerical data were presented as mean ± standard deviation for normal distribution or otherwise median (interquartile range). Categorical data were expressed as number (percentage).

### Chest HRCT characteristics

Patients with and without CRS had a comparable number of bronchiectatic lobes (median: 4.0 vs. 4.0, P = 0.28) and chest HRCT total score (median: 7.0 vs. 7.0, P = 0.46). Intriguingly, patients with CRS had a lower proportion of cystic bronchiectasis (42.6% vs. 61.4%, P = 0.03). The proportion of other HRCT characteristics, including predominantly middle/lower lobe bronchiectasis (76.6% vs. 68.3%, P = 0.30), pulmonary emphysema (19.1% vs. 17.8%, P = 0.85), dyshomogeneity (66.0% vs. 62.4%, P = 0.67), atelectasis (25.5% vs. 27.8%, P = 0.78), pulmonary cavity (38.3% vs. 43.6%, P = 0.55), infiltrations (87.2% vs. 92.1%, P = 0.35) and bilateral bronchiectasis (85.1% vs. 80.2%, P = 0.47), were consistently non-significant. (Table 3)

**Table 3:** Chest imaging characteristics, lung function, sputum bacteriology, sputum inflammatory biomarkers and cough sensitivity.

Parameters	CRS	No CRS	P value
**No. of patients**	47	101	-
**No. of bronchiectatic lobes**	4.0 (3.0)	4.0 (3.0)	0.28
**HRCT total score**	7.0 (7.0)	7.0 (5.0)	0.46
**Cystic bronchiectasis (No., %)**	**20 (42.6%)**	**62 (61.4%)**	**0.03**
**Predominantly middle/lower lobe bronchiectasis (No., %)**	36 (76.6%)	69 (68.3%)	0.30
**Pulmonary emphysema (No., %)**	9 (19.1%)	18 (17.8%)	0.85
**Dyshomogeneity (No., %)**	31 (66.0%)	63 (62.4%)	0.67
**Atelectasis (No., %)**	12 (25.5%)	28 (27.8%)	0.78
**Cavity (No., %)**	18 (38.3%)	44 (43.6%)	0.55
**Pulmonary infiltration (No., %)**	41 (87.2%)	93 (92.1%)	0.35
**Bilateral bronchiectasis (No., %)**	40 (85.1%)	81 (80.2%)	0.47
**Lung function**	-	-	-
** FVC% predicted**	78.2±19.8	82.2 (16.8)	0.54
** FEV**_**1**_ **% predicted**	68.2±24.8	74.8 (21.2)	0.29
** FEV**_**1**_ **/FVC%**	75.3 (19.6)	73.8±12.7	0.28
** MMEF% predicted**	51.2±29.3	59.2±31.0	0.14
** D**_**L**_ **CO% predicted**	89.7±14.1	89.0±19.9	0.84
**Baseline sputum bacteriology (No., %)**	-	-	0.27
** *Pseudomonas aeruginosa***	17 (36.2%)	27 (26.7%)	-
** Other potentially pathogenic microorganisms** *	15 (31.9%)	28 (27.7%)	-
** Commensals**	15 (31.9%)	46 (45.5%)	-
**Sputum bacterial colonization (No.,%)**	-	-	0.12
** *Pseudomonas aeruginosa***	17 (36.2%)	22 (21.8%)	-
** Other potentially pathogenic microorganisms** *	4 (8.5%)	6 (5.9%)	-
** None**	26 (55.3%)	73 (72.3%)	-
**Sputum inflammatory biomarkers**	-	-	-
** Sputum IL-1β (ng/ml)** **	18.6	19.7	0.85
** Sputum TNF-α (ng/ml)** **	10.3	8.3	0.30
** Sputum IL-6 (ng/ml)** **	2.6	2.0	0.14
** Sputum IL-8 (ng/ml)** **	117.3	110.4	0.52
**Capsaicin cough sensitivity**	-	-	-
** C2 (μmol/L)**	3.9 (123.0)	11.7 (123.0)	0.81
** C5 (μmol/L)**	62.5 (996.0)	250.0 (973.0)	0.32

Numerical data were presented as mean ± standard deviation for normal distribution or otherwise median (interquartile range). Categorical data were expressed as number (percentage).

Data in bold indicated the comparisons with statistical significance.

* Other potentially pathogenic microorganisms included *Haemophilus influenzae*, *Haemophilus parainfluenzae*, *Staphylococcus aureus*, *Stenotrophomonas maltophilia*, *Escherichia colitis*, *Sphingomonas paucimobilis*, *Alcaligenes faecalis subsp faecalis*, *Psedumonas pseudoalcaligenes* and *Serratia marcescens*.

** Data were presented as geometric means.

IL: interleukin; TNF: tumor necrosis factor

### Lung function

No notable disparities were found in lung function parameters, including FVC [78.2±19.8% vs. 82.2 (16.8)%, P = 0.54], FEV_1_ [68.2±24.8% vs. 74.8 (21.2)%, P = 0.29], FEV_1_/FVC [75.3 (19.6)% vs. 73.8±12.7%, P = 0.28], MMEF (51.2±29.3% vs. 59.2±31.0, P = 0.14) and D_L_CO predicted (89.7±14.1% vs. 89.0±19.9%, P = 0.84), despite that patients with CRS tended to have greater lung function impairment. (Table 3)

### Sputum bacteriology

Patients with CRS tended to have a non-significantly higher rate of *Pseudomonas aeruginosa* isolation (36.2% vs. 26.7%, P = 0.27) and a lower proportion of commensals isolation (31.9% vs. 45.5%, P = 0.27) at baseline. Furthermore, the proportion of *Pseudomonas aeruginosa* infection (36.2% vs. 21.8%, P = 0.12) was relatively higher and the rate of no potentially pathogenic bacteria colonization (55.3% vs. 72.3%, P = 0.12) was lower in the CRS group. (Table 3)

### Sputum inflammatory biomarkers and cough sensitivity

Patients with CRS had non-significantly increased levels of sputum TNF-α (geometric mean: 10.3ng/ml vs. 8.3 ng/ml, P = 0.30), IL-6 (geometric mean: 2.6ng/ml vs. 2.0ng/ml, P = 0.14) and IL-8 (geometric mean: 117.3ng/ml vs. 110.4ng/ml, P = 0.52), but not IL-1β (geometric mean: 18.6ng/ml vs. 19.7ng/ml, P = 0.85).

Similarly, non-significantly lower levels of C2 [3.9 (123.0) μmol/L vs. 11.7 (123.0) μmol/L, P = 0.81] and C5 for capsaicin challenge test [62.5 (996.0) μmol/L vs. 250.0 (973.0) μmol/L, P = 0.02] were noted in patients with CRS. (Table 3)

### BEs within the previous 2 years and during follow-up

**Fig 1:**
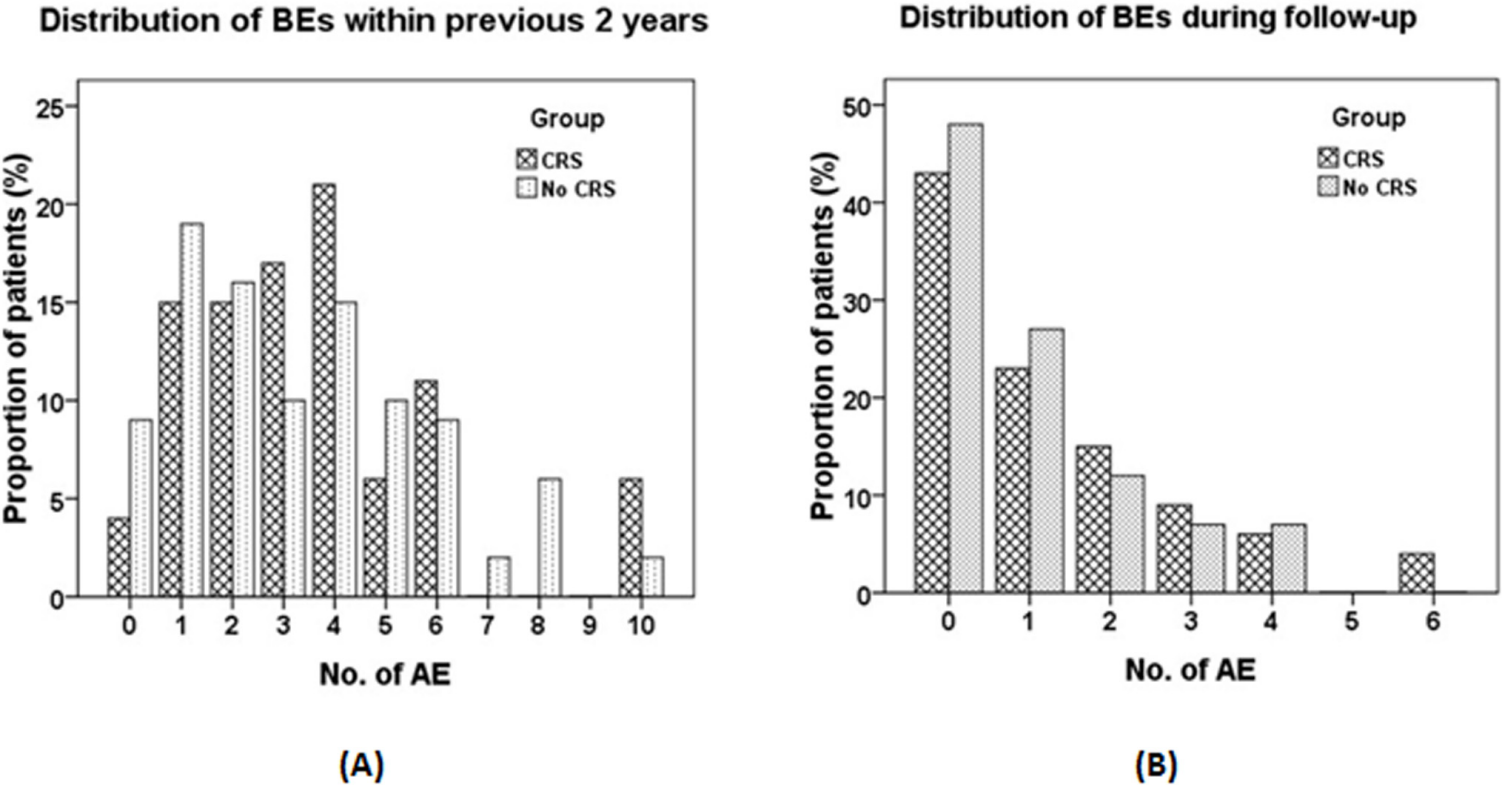
Distribution of the number of BEs within 2 years before enrollment and during follow-up. (A). Distribution of the number of BEs within 2 years before enrollment. Shown are the percentages of patients with different numbers of bronchiectasis exacerbations within the previous 2 years. Light dotted bars indicated the bronchiectasis patients without CRS, whereas the dark cross bars indicated the bronchiectasis patients with CRS. (B). Distribution of the number of BEs during follow-up. Shown are the percentages of patients with different numbers of bronchiectasis exacerbations during the 1-year’s follow-up since patients recruitment. Light dotted bars indicated the bronchiectasis patients without CRS, whereas the dark cross bars indicated the bronchiectasis patients with CRS. AEs: acute exacerbations of bronchiectasis.

(**Fig 1A**) shows the distribution of BEs within 2 years before recruitment. The proportion of patients with different frequency of BEs was overall comparable between CRS and no-CRS group. However, the presence of CRS was associated with non-significantly greater tendency of experiencing any BEs within the previous 2 years before enrollment (OR = 2.47, 95%CI: 0.52–11.76, P = 0.26), which could also be applied even after adjustment with sex, smoking status, the diagnosis of asthma or COPD, and the BSI (all P>0.05, Table 4).

**Table 4:** Association between CRS and the number of BEs within 2 years before enrollment in patients with bronchiectasis.

Variables	Any bronchiectasis exacerbation within 2 years			At least 4 bronchiectasis exacerbations within 2 years		
	OR	95% CI	P value	OR	95% CI	P value
**CRS diagnosis**	2.47	0.52–11.76	0.26	0.86	0.40–1.86	0.71
**Model 1**	2.69	0.56–12.92	0.22	0.81	0.37–1.76	0.59
**Model 2**	2.83	0.58–13.92	0.20	0.74	0.33–1.62	0.45
**Model 3**	2.88	0.58–14.27	0.20	0.73	0.33–1.62	0.44
**Model 4**	2.56	0.51–12.94	0.26	0.70	0.31–1.58	0.39

OR: Odds ratio; 95%CI: 95% confidence interval; BSI: *Bronchiectasis Severity Index*

Model 1: CRS diagnosis + adjustment with sex

Model 2: Model 1 + adjustment with smoking status

Model 3: Model 2 + adjustment with asthma or COPD diagnosis

Model 4: Model 3 + adjustment with the BSI

The distribution of BEs during follow-up is displayed in (**Fig 1B**). Again, similar distribution patterns of BEs were noted between the two groups.

**Fig 2:**
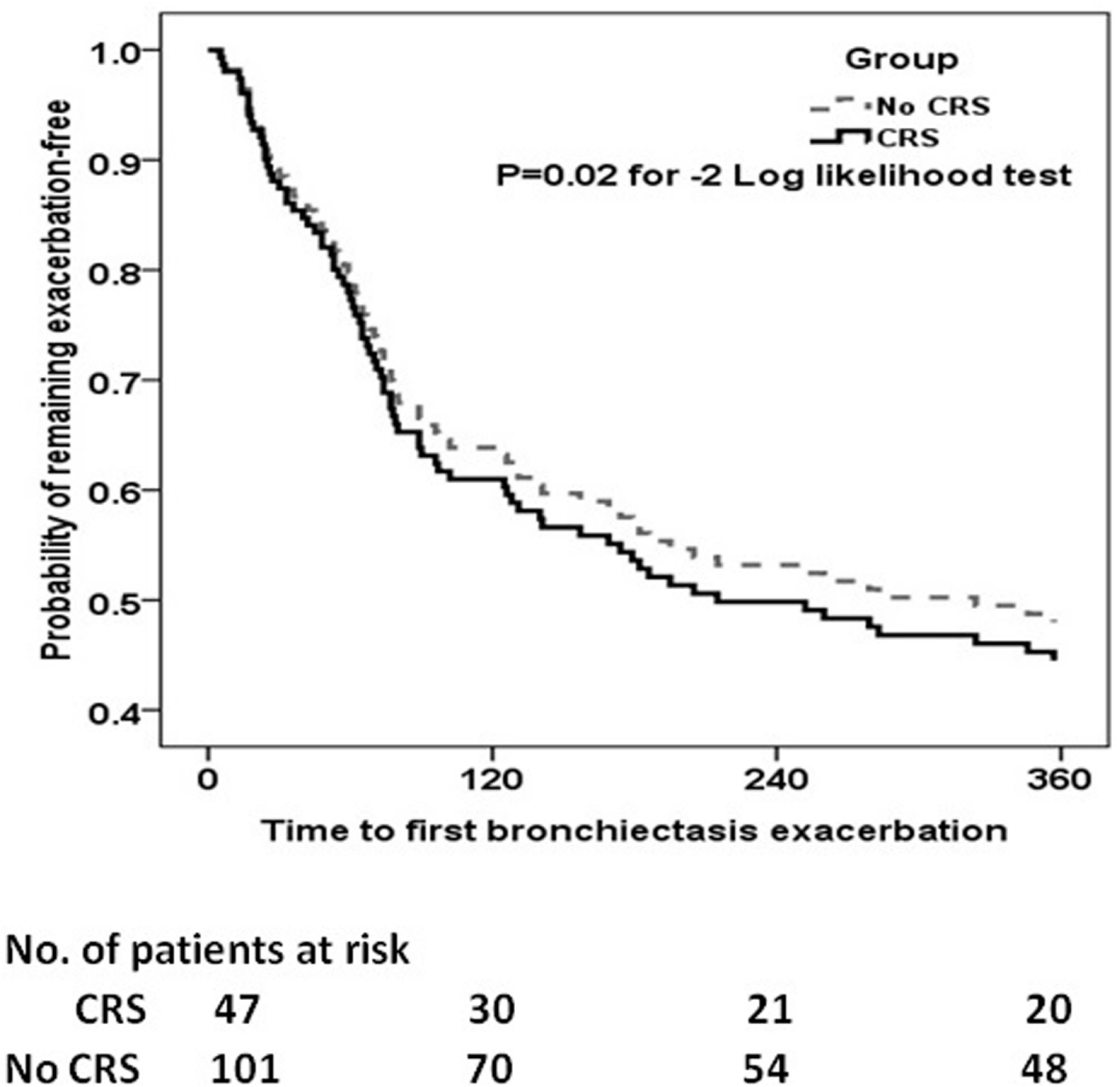
Cox proportional hazards analysis on the risks of having bronchiectasis exacerbations during follow-up in patients with and without CRS. The BEs during follow-up were analyzed with Cox regression model, with sex, smoking status, the diagnosis of asthma or COPD, and the BSI as covariates. The grey dotted line indicated the bronchiectasis patients without CRS, whereas the dark dotted line indicated the bronchiectasis patients with CRS. Shown at the bottom of the figure were the number of bronchiectasis patients at risks of experiencing subsequent exacerbations at different time points.

Following adjustment with sex, smoking status and the BSI, patients with CRS had significantly greater risks of experiencing BEs than those without during the 1-year follow-up period (P = 0.02 for negative binominal regression test, **Fig 2**).

## Discussion

### Principal findings

We have identified a considerable proportion of patients with CRS among the bronchiectasis cohort. Concomitant CRS might be a disease modifier, as indicated by the differences in demographics, chest imaging characteristics, sputum bacteriology, lung function, use of healthcare resources and frequency of BEs before enrollment between the CRS and no-CRS group. However, CRS group had significantly greater risks of experiencing BEs during follow-up, even after adjustment with potential confounders.

### Interpretations

CRS has been shown to confer adverse impacts on patients with bronchiectasis. In a retrospective investigation on 88 bronchiectasis patients (56 idiopathic and 32 post-infectious), Guilemany and associates found that 77% of patients actually had CRS, and that those with CRS presented with considerably higher HRCT score and poorer lung function [2]. A considerable proportion of bronchiectasis patients had loss of smell, suggesting the signs of nasal obstruction [5]. Significant nasal obstruction and airflow limitation were also associated with reduced levels of nasal nitric oxide, particularly in patients with nasal polyps [2]. Furthermore, patients with CRS had significantly worse quality of life, as measured by the St. George’s Respiratory Questionnaire, Sinonasal Outcome Test-20 and Short Form-36 [3]. These offered the clues that CRS conferred the adverse impacts which were not merely restricted to the upper airways.

Our findings were partially inconsistent with previous studies [2–5]. A possible explanation was the relatively lower prevalence of CRS in our Chinese cohort (idiopathic: ~50%, post-infectious: ~30%), which mirrored the experience of the author’s routine practice, despite the lack of epidemiological profiles in mainland China. The higher prevalence of CRS (77%) in Guilemany et al’s serial studies [2–5] might have rendered the adverse impacts more readily displayed. Second, our baseline data were sampled when clinically stable (including sinonasal symptoms), thus the remission of CRS might have tempered the effects on lower airways. It should also be recognized that the presence of CRS in bronchiectasis depends on the etiology, which might help explain the phenomena such as the lower proportion of cystic bronchiectasis seen in bronchiectasis with CRS and the greater proportion patients who had ever been treated with inhaled steroids. Third, the prevalence of nasal polyps was unclear in our study because HRCT alone could not be applied to diagnose or rule out CRS clinically. In Guilemany et al’s study, 21% of patients were found to have concomitant nasal polyps, which further accounted for the greater illnesses as observed in the CRS group. The lack of significant associations between CRS and bronchiectasis might be partially interpreted by the fact that our cohort suffered from milder forms of CRS. Another explanation would have been the minor impacts of CRS on bronchiectasis due to the younger age of our patient cohort, as compared with that in the study by Guilemany et al [2–5].

Intriguingly, our results showed that patients with CRS had a lower proportion of cystic bronchiectasis. A possible interpretation would have been the lower proportion of post-infectious bronchiectasis in patients with CRS. Post-infectious bronchiectasis has often been associated with more exuberant radiological forms, like cystic bronchiectasis. Alternatively, this finding might have been confounded by the proportion of other aetiologies that also elicited cystic bronchiectasis.

Nonetheless, despite the overall negative results between the two groups, we found a trend towards non-significantly higher *BSI*, more frequent BEs needing hospitalization prior to enrollment, poorer lung function (except for diffusing capacity), a higher prevalence of *Pseudomonas aeruginosa* isolated or colonized in sputum, greater capsaicin cough sensitivity and a tendency of more frequent BEs during follow-up. We therefore could not totally preclude the greater adverse impacts of CRS on bronchiectasis, as documented by Guilemany and associates [2–5].

It should be noted that CRS has been traditionally associated with primary ciliary dyskinesia; however, other bronchiectasis aetiologies might also be related to the presence of CRS. For instance, in the study by Guilemany et al [2], 77% of patients (64% had post-infective and 36% had idiopathic bronchiectasis) were diagnosed as having CRS, which indicated that the aetiologic spectra of patients with CRS mirrored those of the whole bronchiectasis cohort. Therefore, one might be unlikely to anticipate a high rate of primary ciliary dyskinesia in bronchiectasis patients with CRS in our cohort. Nonetheless, we do speculate that certain ciliary defects or aberrant beating patterns might exist in this population. Further studies addressing this issue are of merit.

### Clinical implications

Currently, it is unclear whether there would be major ethnic differences in the prevalence of CRS and its impacts on bronchiectasis. However, the findings in Caucasians were unlikely to be suitable for direct translation into the oriental population such as the Chinese patients. Physicians should be vigilant to the co-existence of CRS in bronchiectasis patients, particularly CRS with nasal polyps. Whether active treatment of upper airway symptoms would lead to substantial amelioration in the lower airway symptoms remains doubtful, but it is likely that proper management of CRS should at least improve the quality of life, sense of smell and post-nasal drip symptoms in bronchiectasis. Furthermore, the advantage of proper treatment of CRS will be the prevention of infection or re-infection of the tracheobronchial tree, as has been documented in cystic fibrosis. In view of the currently limited therapeutic options [16–23], future clinical trials with ample sample sizes that address these concerns should be given sufficient priority.

Furthermore, irrespective of the impacts of CRS on bronchiectasis, there has been a significant gap between treatment of CRS and the ideal therapeutic outcomes in bronchiectasis. Little attention has been paid to the management of upper airway symptoms in bronchiectasis, a disease in which “one airway, one disease” also applied. Our finding raises physician’s awareness of screening for CRS upon the diagnosis of bronchiectasis, considering proper treatment of CRS might improve the outcomes of bronchiectasis. It is likely that CRS may, despite a lower prevalence compared with western countries, also confer considerable adverse impacts on Chinese patients with bronchiectasis.

### Strengths and limitations

Our study is the first report focusing on the impacts of CRS on bronchiectasis in oriental Asian population. The detailed clinical assessments have enabled the authors to systematically evaluate the magnitude of effects of CRS on bronchiectasis, which might be the basis for future intervention of upper airway symptoms in bronchiectasis patients with CRS.

Nonetheless, there are some concerns that should be highlighted.

** Apart from the comparatively lower prevalence, the imbalanced sample size between the two groups might have also obscured the adverse impacts of CRS.

** The lack of assessment of nasal polyps by using nasal endoscopy and tissue biopsy and the degree of CRS (based on visual analog scales) has precluded further analyses on the association between the severity of CRS and that of bronchiectasis.

** Nasal nitric oxide was not measured, which has limited the capacity for examining ciliary defects in our cohort.

** Furthermore, it should be admitted that cystic fibrosis could not be totally ruled out. However, the recent findings of Liu et al [28] strongly indicated that cystic fibrosis would be unlikely to be diagnosed by simply applying the current international diagnostic standards, and that the oriental population harbors unique and rare gene loci that need to be further investigated. The prevalence of cystic fibrosis was also reportedly very low in Asian countries. Therefore, even if we routinely screened for cystic fibrosis, one might be unlikely to anticipate the identification of many cases in the oriental population.

** Nasal culture was not performed in this study, therefore we were unable to comment on the correlation between nasal microbiota compositions and bronchiectasis exacerbations.

** Finally, nasal mucosa has not been sampled. Therefore, the authors were unable to comment on the association between upper and lower airways inflammation in bronchiectasis patients with and without CRS.

## Conclusion

Chinese adults with bronchiectasis seem to have a lower prevalence of CRS than that reported previously. Despite the lack of statistical significance, there is a trend towards greater disease severity of bronchiectasis, poorer lung function, higher prevalence of *Pseudomonas aeruginosa* isolation or infection, greater capsaicin cough sensitivity and more frequent BEs in patients with CRS.

In future clinical practice, physicians should be vigilant to the screening of concomitant CRS in bronchiectasis so as to better improve patient’s healthcare. Our findings may be of clinical significance in that proper treatment of upper airway symptoms due to CRS will be the prevention of infection or re-infection of the tracheobronchial tree, which should be addressed for the future management of bronchiectasis.
